# Mapping the Development of Human Spermatogenesis Using Transcriptomics-Based Data: A Scoping Review

**DOI:** 10.3390/ijms25136925

**Published:** 2024-06-25

**Authors:** Lena Kwaspen, Marc Kanbar, Christine Wyns

**Affiliations:** 1 Laboratoire d’Andrologie, Pôle de Recherche en Physiologie de la Reproduction, Institut de Recherche Expérimentale et Clinique (IREC), Université Catholique de Louvain, 1200 Brussels, Belgium; lena.kwaspen@uclouvain.be (L.K.); marc.kanbar@uclouvain.be (M.K.); 2 Department of Gynecology-Andrology, Cliniques Universitaires Saint-Luc, 1200 Brussels, Belgium

**Keywords:** scRNA-seq, spermatogenesis, development, transcriptomics, in vitro maturation, germ cells, somatic cells, testicular tissue

## Abstract

In vitro maturation (IVM) is a promising fertility restoration strategy for patients with nonobstructive azoospermia or for prepubertal boys to obtain fertilizing-competent spermatozoa. However, in vitro spermatogenesis is still not achieved with human immature testicular tissue. Knowledge of various human testicular transcriptional profiles from different developmental periods helps us to better understand the testis development. This scoping review aims to describe the testis development and maturation from the fetal period towards adulthood and to find information to optimize IVM. Research papers related to native and in vitro cultured human testicular cells and single-cell RNA-sequencing (scRNA-seq) were identified and critically reviewed. Special focus was given to gene ontology terms to facilitate the interpretation of the biological function of related genes. The different consecutive maturation states of both the germ and somatic cell lineages were described. ScRNA-seq regularly showed major modifications around 11 years of age to eventually reach the adult state. Different spermatogonial stem cell (SSC) substates were described and scRNA-seq analyses are in favor of a paradigm shift, as the A_dark_ and A_pale_ spermatogonia populations could not distinctly be identified among the different SSC states. Data on the somatic cell lineage are limited, especially for Sertoli cells due technical issues related to cell size. During cell culture, scRNA-seq data showed that undifferentiated SSCs were favored in the presence of an AKT-signaling pathway inhibitor. The involvement of the oxidative phosphorylation pathway depended on the maturational state of the cells. Commonly identified cell signaling pathways during the testis development and maturation highlight factors that can be essential during specific maturation stages in IVM.

## 1. Introduction

Human spermatogenesis is a process involving the transition from diploid spermatogonial stem cells (SSCs) to haploid fertilizing-competent spermatozoa [1,2,3]. While SSCs are life-long-present cells in the testis, differentiating spermatogonia exist solely for one spermatogenic cycle, whereafter they maturate into primary spermatocytes [4,5]. These cells undergo the first meiosis to become secondary spermatocytes, which develop into round spermatids after the second meiosis, and these round spermatids undergo spermiogenesis to finally maturate into spermatozoa [4]. During this process, round spermatids develop into elongated spermatids, where processes of acrosome development and acquisition of cell’s structure required for mobility and nucleus condensation following DNA packaging with histone-protamine replacement, take place [1,2,4]. Elongated spermatids then transition into spermatozoa after being released from Sertoli cells (SCs) [4]. The cells of the germ cell lineage are supported by cells of the somatic cell lineage, such as SCs, Leydig cells (LCs), and peritubular myoid cells (PTMCs) [6]. Although spermatogenesis was discovered more than 150 years ago, the complete human process could not be reproduced in vitro [7] despite the many attempts since the work of Steinberger et al. in 1984 [8]. Efforts were increased during the last decades to identify suitable culture systems and media [9,10,11,12,13,14,15,16] in response to more recent needs. In addition to the treatment of nonobstructive azoospermia due to maturation arrest of the germ cells (GCs), in vitro maturation is also an option to restore the fertility of childhood survivors of hematological or metastasizing malignancies who preserved their immature testicular tissue (ITT) prior to gonadotoxic therapies [17,18]. Patients suffering from benign hematological conditions requiring hematopoietic stem cell transplantation [19], and patients with disorder of sex development who preserved their testis tissue for potential future use at the time of bilateral orchiectomy [20] could also benefit from this approach. While in vitro maturation (IVM) of nonhuman ITT has already been achieved in a variety of species [18] with healthy offspring in rodents [21,22], the highest achievements with human ITT were the development of fertilization-competent spermatids starting from fetal tissue [23], and the development of round spermatids from prepubertal tissue, although in very limited amounts [15]. For nonobstructive azoospermic patients, in vitro culture of SSCs inside their testicular niche up to the post-meiotic state were also reported [24]. The next step towards the development of a clinically applicable IVM method for human testicular tissue is to increase knowledge on the maturation and differentiation processes of the different testicular cell types from the neonatal to the post-pubertal period. Gene expression data deriving from single-cell RNA-sequencing (scRNA-seq) experiments is very informative to analyze and understand the development of the different testicular cell types over different ages.

This scoping review aims to frame the development and maturation of the testis by summarizing data from research papers containing original scRNA-seq data of the native human testis from the fetal period towards late adulthood. Gaining a better understanding of this process presents the ultimate goal of reproducing it in vitro. Additionally, efforts to compare this data with scRNA-seq on cultured testicular cells were made in an attempt to identify dysregulations and potential new targets to optimize IVM of human testicular tissue.

## 2. Materials and Methods

### 2.1. Study Design

This is a scoping review with a published protocol, written via the PRISMA guidelines for protocols, in Open Science Framework network with the latest update on the 25 March 2024 (https://osf.io/8xrbv/) [25].

### 2.2. Literature Search

The databases PubMed, Embase, and the GEO DataSet were screened for articles from 2009 to 31 March 2024. Papers published before 2009 were excluded because this was the year that scRNA-seq was published for the first time [26]. The literature search was conducted by combining the following concepts: “testis” and “Single-Cell Gene Expression Analysis” in humans. Forward and reverse snowballing was allowed to identify extra articles of interest. The full literature search strings for the three databases are included in Appendix A, Appendix B and Appendix C.

### 2.3. Eligibility Criteria

The inclusion criteria were that the full text of the original research paper was available in English and scRNA-seq was performed on human testicular tissue samples, containing seminiferous tubules. For papers that reported a comparison between normal and diseased tissue, data from the control group were included.

Papers on samples from post-pubertal patients with a known abnormality of spermatogenesis and studies that did not mention the age/maturational status of the testicular tissue or reanalyzed published data were excluded.

### 2.4. Study Selection

**Figure 1:**
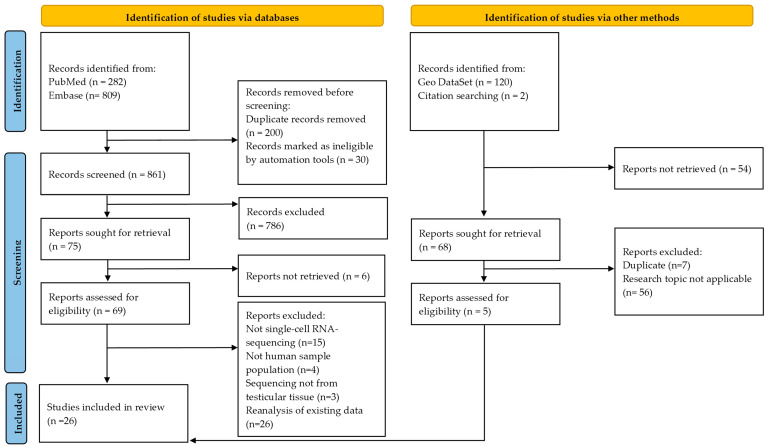
PRISMA flowchart [27] of screened papers including the numbers of identified references, the numbers and reasons for exclusion, and final total number of included studies.

The title and abstract screening were performed by K.L. and K.M. separately, independently, and in a blinded way. The two searches were compared and discussed between K.L. and K.M., and in all cases, consensus was reached after the involvement of all the authors. The PRISMA flowchart illustrating the selection process of identified references is illustrated in Figure 1.

### 2.5. Data Extraction and Interpretation

Full-text screening was performed separately, independently, and blinded by K.L. and K.M. Endnote (v.21.0.1) and an Excel file (Microsoft, v2311 Build 16.0.17029.20178) were used to process the full-text screening. Where needed, the results of the blinded, individual screening process were discussed among all authors.

The data that were extracted consisted of the name of the first author, the year of publication, the GEO series accession (GSE) number or other databases where the data was made available, identified cell types, the number of participants and their age, interesting information regarding the spermatogonia, spermatocytes, spermatids, spermatozoa, Leydig cells, Sertoli cells, peritubular myoid cells, and macrophages, as well as the culture time, and added supplements if applicable.

The cells from the GC lineage from prenatal samples are labeled in this review paper as GCs, and from the neonatal phase onwards, these cells are called SSCs due to the variety of reported cell names between different papers.

The synthesis of the results is provided in a narrative, descriptive format.

## 3. Results and Discussion

**Figure 2:**
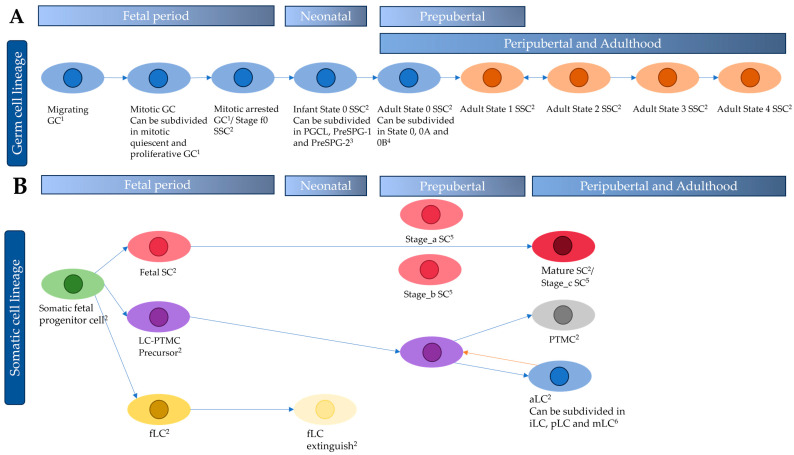
Overview of the germ cell lineage development and the development of the SC, PTMC, and LC in the somatic cell lineage. The fetal period is between 4 and 25 weeks post-conception, the neonatal period is between 2 days after birth and 5 months of age, the prepubertal period is from 1 to 11 years old, the peripubertal period is between 13 and 14 years old, and adulthood contains samples between 17 and 55 years old and 60 and 90 years old. (**A**) In the germ cell lineage, the blue color represents the differentiation in the fetal period and the postnatal maturation of the State 0 SSC, the brown SSCs represent the maturation of the State 0 SSC of the more differentiated SSC states. Names of the GCs are given based on the study of Li et al. ^1^ [30]. SSCs, SCs, LC, LC-PTMC precursor, somatic fetal progenitor cell, and the PTMC are defined based on the studies of J. Guo et al. ^2^ [31,36,38]. The neonatal subdivision of the infant SSC state is based on the study results of Sohni et al. ^3^ [34], and the subdivision of the adult State 0 SSC is based on Voigt et al. ^4^ [35]. (**B**) In the somatic cell lineage, the different colors represent different cell states. The different SC states in the pre- and peripubertal phases are named according to the study of Zhao et al. ^5^ [37]. Mahyari et al. ^6^ identified in the aLC the iLC = immature LC, pLC = progenitor LC, and mLC = mature LC [48]. The orange line indicates the dedifferentiation of the aged LCs [49]. Abbreviations: GC = germ cell, SSC = spermatogonial stem cell, SC = Sertoli cell, fLC = fetal Leydig cell, aLC = adult Leydig cell, LC-PTMC precursor = Leydig-peritubular myoid cell precursor, PTMC = peritubular myoid cell.

In the literature search, scRNA-seq studies were found for both native tissue and a part was performed on cultured testicular cells. In total, 1091 articles were identified from PubMed, Embase, and Gene Expression Omnibus (GEO) DataSet. Twenty-six studies fulfilled the eligibility criteria and were included in this scoping review (Figure 1). Among these studies, except the 2 that only studied the transcriptome after cell culture, 15 studies included a single age category and 9 two age categories, enabling us to present the results on native tissue according to the different life stages. ScRNA-seq data were identified for 69 prenatal samples with an age between 4 and 25 weeks post-conception (WPC) [28,29,30,31,32,33], 2 neonatal samples from 2 to 7 days old [34], and 1 of 5 months old [31], 11 testis samples from prepubertal patients of 1 to 11 years old [35,36,37,38], 2 peripubertal samples of 13- and 14-year-old boys [36], 53 testicular samples from adults between 17 and 55 years old [5,34,37,38,39,40,41,42,43,44,45,46,47,48,49,50], and 14 testis samples derived from patients between 60 and 90 years old [5,42,49,50]. Table 1 shows the research papers analyzing native testicular tissue that fulfilled the eligibility criteria classified over the different developmental stages. Publications containing samples of different developmental stages are reported multiple times in the table, with, for each of the developmental stages, the number of newly sequenced samples mentioned separately. Figure 2 shows the timeline of the differentiation and maturation of the GC lineage and the major cell types in the somatic cell lineage for each developmental period. Moreover, three papers reporting scRNA-seq on cultured testicular cells were identified and are listed in Table 2. In these papers, one paper cultured fetal testicular cells, one cultured adult SSCs, and another cultured adult PTMCs.

### 3.1. Fetal Testis

#### 3.1.1. The Germ Cell Lineage

ScRNA-seq identified three GC states, which were mainly present in the fetal testis. The different GC states were the migrating GCs, the mitotic GCs that can be subdivided into the mitotic quiescent and mitotic proliferative state, and the mitotic arrested GCs (Figure 2). The migrating GCs start to migrate into the gonadal ridge around 4 WPC [30,51] and the gene expression of these GCs could be identified in the scRNA-seq data of Li et al. Gene Ontology (GO) terms that describe the biological processes, molecular functions, or cellular components to support the interpretation of the biological function of the related genes [52] were recorded. The three most significant GO terms in these GCs were “generation of precursor metabolites and energy”, “electron transport chain”, and “oxidative phosphorylation” [30]. F. Guo et al. determined three other most significant GO terms from their transcriptomics results: “cytoskeleton organisation”, “chromosome organisation”, and “cell division” [32].

From 8 to 10 WPC, mainly mitotic quiescent GCs were present in the fetal testis and slightly more than 25% of the mitotic GCs were in the proliferative state [29]. According to Li et al., the three most significant GO terms for the overall mitotic GCs were “translational elongation”, followed by “translation” and “DNA damage response, signal transduction” [30]. F. Guo et al. identified GO terms for GCs of 10 WPC as “response to DNA stimulus”, “cellular response to stress”, “cell division”, and “chromatin modification” [32].

After 10 WPC, scRNA-seq results showed that the fraction of the mitotic arrested GCs started to increase from less than 10% to more than 60%, which then became the most abundant cell state at 23 WPC [29]. Li et al. could identify a small population of cells in a transition state, which had a gene expression pattern similar to both the mitotic GC state and the mitotic arrest GC state. Furthermore, the mitotic arrest state is marked, according to Li et al., with GO terms such as “gamete generation”, “transcription”, “sexual reproduction”, “regulation of transcription”, and “negative regulation of transcription from RNA pol II promotor” [30]. F. Guo et al. found overlapping GO terms as “sexual reproduction” and “gamete generation”, but also identified “spermatogenesis”, “chromosome organisation”, “meiosis”, and “multicellular organism reproduction” [32]. In addition, Li et al. found that 17 cell-cycle-arrest-related genes were upregulated in the mitotic arrest GC state. Among these, eight genes (NANOS2, CDKN2B, TCF7L2, CDK6, PCBP4, NFATC1, SESN3, PDK1) were specific to the mitotic arrest GC state, one gene (STK11) was also expressed in the mitotic proliferative GC state, and eight genes (CGRRF1, NBN, CUL3, CUL4A, DEK, PHGDH, MLF1, KIF20B) were found in all GC states [30].

One of the studies by J. Guo et al. identified that these mitotically arrested cells (described as fetal State 0 or State f0) did not express pluripotency hallmarks anymore but already expressed SSC markers (PIWIL4, EGR4, MSL3, and TSPAN33). These results suggested that the State f0 SSC was already the precursor of the undifferentiated neonatal and adult SSC pool (State 0) [31].

Moreover, the group of R. Wang et al. was able to identify in their scRNA-seq data an additional GC type, SPARC positive cells, which were detected in fetal samples from 6 to 23 WPC [29]. SPARC has an important function in the migration of cells [53], indicating that these cells were possibly able to migrate in the gonads [29].

**Table 1:** Research articles that analyzed native testicular tissue and fulfilled the eligibility criteria.

Developmental Stage	Author	Year of Publication	Number of Participants	Age	GSE Number	Other Database	Reference
Prenatal	F. Guo et al.	2015	15	4–19 WPC	GSE63818		[32]
	Li et al.	2017	12	4–25 WPC	GSE86146		[30]
	Chitiashvili et al.	2020	5	6–16 WPC	GSE143356	http://germline.mcdb.ucla.edu (accessed on 26 March 2024)	[33]
	J. Guo et al.	2021	6	6, 7, 8, 12, 15, 16 WPC	GSE143356		[31]
	Garcia-Alonso et al.	2022	22	6–21 WPC		https://www.reproductivecellatlas.org/ (accessed on 26 March 2024)	[28]
	R. Wang et al.	2022	9	6–23 WPC			[29]
Neonatal	Sohni et al.	2019	2	2, 7 days	GSE124263		[34]
	J. Guo et al.	2021	1	5 months	GSE161617		[31]
Prepubertal	J. Guo et al.	2018	2	1 year	GSE120508		[38]
	J. Guo et al.	2020	2	7, 11 years	GSE134144	https://humantestisatlas.shinyapps.io/humantestisatlas1/ (accessed on 26 March 2024)	[36]
	Zhao et al.	2020	4	2, 5, 8, 11 years	GSE149512		[37]
	Voigt et al.	2022	3	1, 2, 7 years	GSE196819		[35]
Peripubertal	J. Guo et al.	2020	2	13, 14 years	GSE134144		[36]
Adult	J. Guo et al.	2017	5	adult	GSE92280		[39]
	Neuhaus et al.	2017	5	adult	GSE91063		[40]
	J. Guo et al.	2018	3	17, 24, 25 years	GSE120508		[38]
	Hermann et al.	2018	7	37, 38, 34, 36, 49, 43, 43 years	GSE108977, GSE109037	https://data.mendeley.com/datasets/kxd5f8vpt4/1 (accessed on 26 March 2024)	[41]
	M. Wang et al.	2018	1	30 years	GSE106487		[42]
	Sohni et al.	2019	2	37, 42 years	GSE124263		[34]
	Chen et al.	2020	1	36 years	GSE144085		[43]
	Shami et al.	2020	4	20–40 years	GSE142585		[44]
	B. Xia et al.	2020	2	40, 45 years	GSE125372		[45]
	Zhao et al.	2020	6	17, 23, 25, 28, 28, 31 years	GSE149512		[37]
	Alfano et al.	2021	1	37 years	GSE154535		[46]
	Di Persio et al.	2021	3	31, 33, 55 years	GSE153947		[47]
	Mahyari et al.	2021	4	adult	GSE169062	https://github.com/eisascience/HISTA and https://doi.org/10.5281/zenodo.4433041 (accessed on 26 March 2024)	[48]
	Nie et al.	2022	4	17–22 years	GSE182786		[49]
	K. Xia et al.	2022	3	28, 24, 31 years			[5]
	Gui et al.	2024	2	22, 30 years		Data available on request	[50]
Elderly	M. Wang et al.	2018	1	60 years	GSE106487		[42]
	Nie et al.	2022	8	62–76 years	GSE182786		[49]
	K. Xia et al.	2022	3	61, 70, 87 years			[5]
	Gui et al.	2024	2	80, 90 years		Data available on request	[50]

WPC = weeks post-conception, GEO series accession (GSE) number [54].

In addition to this, Chitiashvili clustered GCs (4–16 WPC-old) [33], based on their gene expression [55], and found 5 different GC clusters. They also reanalyzed the data of Li et al. and found 7 cell clusters. Unfortunately, the cell classification of migrating, mitotic, and mitotic arrested GC type was not allocated to the different clusters [33].

During the fetal developmental period, an upregulation of the base–excision repair (BER) pathway was found in the GCs. The BER pathway participates in the demethylation of DNA, which is confirmed by the results of the DNA methylome analysis that showed a decrease in methylated DNA over time in GCs of 7–19 WPC [32]. Additional pathways were identified, such as the bone morphogenetic protein (BMP) pathway, which has a role in the differentiation of GCs [29,30]. Furthermore, expression of the NODAL ligands for the NODAL signaling pathway was identified in the mitotic GCs and the corresponding receptor in the mitotic arrest GCs. The transforming growth factor-β signaling pathway was involved in the mitotic arrest GC state and the KIT pathway and the NOTCH pathway were activated in the mitotic GCs and mitotic arrested GCs [30].

#### 3.1.2. The Somatic Cell Lineage

ScRNA-seq data confirmed that the proportion of cells from the somatic lineage is remarkably larger than the germ cell lineage in the prenatal testis [29,31]. Although the proportion of somatic cells is larger, the average amount of gene expression, which is between 50,900 and 147,000 copies of mRNA per somatic cell, is lower than the average gene expression of 94,200 to 279,300 copies of mRNA per GC, as reported in the 4–19 WPC testis [32].

A heterogeneous, common, fetal progenitor cell was identified around 6–8 WPC, and scRNA-seq data showed that this cell splits into the SC lineage, fetal LCs (fLCs), and the Leydig-peritubular myoid cell (LC-PTMC) precursors [31].

Garcia-Alonso et al. sequenced 6–21 WPC testicular tissues and was able to identify two resident, testis-specific macrophage populations. The first group of macrophages had an osteoclast-like signature with an assumed role in the promotion of mesonephric endothelial cell (EC) migration, which is required for the testis cord formation. These cells could be found in the interstitial space close to the ECs. The second set of macrophages expressed a microglia-like structure and was found inside the testis cords, potentially having a role in the phagocytosis machinery, immune response, and cell communication between SCs and GCs [28].

### 3.2. Neonatal Testis

#### 3.2.1. The Germ Cell Lineage

From the prenatal to the neonatal period, the mitotically arrested GCs (or State f0 SSCs) developed into an infant State 0 SSCs [31]. In addition to the latter study, Sohni et al. discovered three consecutive cell states in the neonatal testis. The first group were cells with a closely related expression profile to GCs; therefore, they were named as primordial GC-like cells, and the following consecutive prespermatogonia (PreSPG) subtypes were named PreSPG-1 and PreSPG-2 and were likely the precursors of the adult SSC population [34].

#### 3.2.2. The Somatic Cell Lineage

During the first month of life, fLCs could still be detected in the scRNA-seq data of J. Guo et al., although fLCs were not present anymore in the testis of a five-month-old boy. Additionally, the LC-PTMC precursors were still detected [31], as shown in Figure 2.

#### 3.2.3. Cell Interaction

Cell–cell interactions are enabled via paracrine factors to regulate the cell development [56]. The scRNA-seq data revealed that the NOTCH pathway enabled cell communication between the neonatal GCs and the SCs. It was suggested that the WNT pathway makes germ cell–somatic cell communication and somatic cell–somatic cell communication possible. It was observed that the WNT ligand (RSPO2) and receptor (LGR4) were expressed by the neonatal GCs and SCs. Another WNT ligand (WNT6) and the receptor (FRZB) were both expressed in SCs and PTMCs. The data showed that via the KIT pathway SCs and ECs possibly communicated with neonatal GCs and especially the preSPG-2 cells. Similarly, the SCs upregulated the DHH ligand of the Hedgehog signaling pathway, whose receptor was expressed in the neonatal GCs and LCs [34].

### 3.3. Prepubertal and Peripubertal Testis

#### 3.3.1. The Germ Cell Lineage

J. Guo et al. identified five different cell states in the SSC population (State 0–4) via cell clustering and pseudotime trajectory analysis [36,38], which is used to predict the differentiation or maturation of cells via allocating cells according to similarities on a trajectory [57].

In prepubertal boys of 1–7 years old, only quiescent or slowly-self renewing undifferentiated SSCs were present, which were subdivided into State 0 and State 1 SSCs. The differentiating spermatogonia (States 2 to 4) as well as the spermatocytes and, rarely, some spermatids could already be identified in the testis at the age of 11 years [36].

Voigt et al. studied the metabolic fingerprints in the undifferentiated SSC pool of testicular tissue samples and found that genes related to hypoxia were only upregulated in SSCs during the first year of life, whereafter the expression declined. Oxidative phosphorylation (OXPHOS) and several related ATP-ase genes were also upregulated in the first year after birth but stayed about the same expression level for 10 more years before being downregulated. Simultaneously with the downregulation of OXPHOS, the hypoxia pathway was again temporarily upregulated. These changes at the gene level (found in the sample of an 11-year-old patient) are accompanied by a morphological change of the undifferentiated SSCs from round to flat, as evidenced by immunofluorescent staining. Voigt et al. also studied other pathways and detected between the ages of 1 and 2 years an enrichment of the tumor necrosis factor-signaling-via NFκB, interferon-γ response, interferon-α response, and p53-pathways in SSCs, and this was also true between the ages of 11 and 14 years. The IL6/JAK/Stat3 and IL2/Stat 5 signaling pathways were only enriched in the 2-year-old SSCs compared to the 1-year-old. In addition, the reactive oxygen species pathway was slightly upregulated between the age of 1 and 2 years and between 11 and 14 years, and the KRAS signaling pathway was also a little bit upregulated at the ages of 1–2 years and 14–17 years [35].

#### 3.3.2. The Somatic Cell Lineage

The maturation of juvenile SCs was extensively investigated by two research groups. On the one hand, J. Guo et al. sequenced 1-, 7-, 11-, 13-, and 14-year-old samples and named the different discovered SC states: Immature#1, Immature#2, and Mature [36]. On the other hand, Zhao et al. used samples of 2-, 5-, 8-, 11-, and 17-year-old patients to determine the three distinct SC states: Stage_a, Stage_b, and Stage_c [37]. In this review, the different states were appointed as in the paper of Zhao et al., due to the more “neutral” designation. Stage_a SCs were the major cell state at the ages of 1 and 2 [36,37]. The Stage_a SC has, in contrast to the Stage_b SC, a high expression of the androgen receptor target genes [36]. Zhao et al. found that Stage_a SCs express more mitotic genes than Stage_b SCs [37], although the difference in mitotic genes expression was not seen between the different SC stages in the data of J. Guo et al. [36]. The proportion of the Stage_b SC population was increasing with time [36,37], and from 11 years onwards, Stage_c SCs were already identified in the study of J. Guo et al. [36], whereas Zhao et al. determined Stage_c cells starting from the age of 17 [37].

The LC-PTMC precursors stayed present in the prepubertal period (1–11 years) [31,36], and this cell type was characterized by the GO terms: “response to stimulus”, “secretion”, and “transcription regulation”. Furthermore, the three cell types were detected in a 13-year-old boy (LC-PTMC precursors, aLCs, and PTMCs). In a 14-year-old testis, the LC-PTMC precursor population ceased to exist, the cell lineages were completely segregated, and the separated cell lines persisted during adulthood. ScRNA-seq data showed that the PTMCs expressed genes related to cell adhesion and cytoskeleton, in contrast to aLCs, which expressed secretion-related genes for testosterone production [36].

### 3.4. Adult Testis

The scRNA-seq determined that the cell proportions in adult testicular tissue contained 39% spermatogonia, 6% spermatocytes, 1% spermatids, 2% LCs, 24% PTMCs, 1% macrophages, and 27% ECs and blood cells together (SC number was not reported) [34].

#### 3.4.1. The Germ Cell Lineage

SSCs were a heterogenous cell population [40,44]. From the five described SSC states, State 0 and State 1 SSCs are the undifferentiated spermatogonia [36,38], which are responsible for the lifelong spermatozoa production in the testis [58]. According to the results of J. Guo et al., both states contained GO terms such as “phosphoprotein”, “transcription”, and “protein binding” [38]. The State 1 SSC maturates further into differentiating spermatogonia States 2, 3, and 4 [38,49]. In addition to the previous study, Sohni et al. identified four SSC states, such as the SSC-1 and SSC-2 cell states, which were mitotic quiescent or slow-proliferating, and two differentiating spermatogonia cell states. GO terms reported for SSC-1 were “translational initiation”, “SRP-dependent cotranslational protein targeting to membrane”, and “nuclear-transcribed mRNA catabolic process, nonsense-mediated decay”. For SSC-2, GO terms such as “transcription, DNA-template” and “negative regulation of transcription from RNA pol-II promotor” were identified. The SSC-1 could further be divided into three substates: SSC-1A, SSC-1B, and SSC-1C, where SSC-1B was the most undifferentiated SSC state. SSC-1B maturated into SSC-1A or SSC-1C to become the SSC-2, which mainly expressed genes for cellular differentiation and transcription [34].

In addition to this, Di Persio et al. also subdivided the State 0 SSCs into three different substates: State 0, State 0A, and State 0B [47], and found that all these different cell substates mostly correlated with the State 0 reported in J. Guo et al. [38] and with the three substates of Sohni et al.: SSC1-B, SSC1-A, and SSC1-C, respectively [34]. Furthermore, Shami et al. could identify four different SSC states (hSPG1–4), which highly correlated to the States 0–4 reported in J. Guo et al. [38,44]. Three teams did not report different undifferentiated SSC substates, although they were able to distinguish between undifferentiated SSCs and differentiating spermatogonia [41,42,45].

Di Persio et al. and Guo et al. both performed RNA velocity analyses [38,47], which is an approach to computationally determine a vector from individual cells that predicts the direction of differentiation or maturation towards the next developmental state [59]. It was shown that State 0 of J. Guo et al. had two subpopulations, where one subpopulation was progressing towards State 1 and the other was not [38]. A similar result was seen in the transcriptional trajectory of Di Persio et al., showing that among the three State 0 substates (0, 0A, and 0B), State 0B progressed towards State 1 [47]. Remarkably, both studies found that the State 2 SSC did not only develop towards State 3, but at the same time a reverse progression from State 2 SSC towards State 1 was observed [38,47]. These results indicated that SSC subpopulations were dynamic and presented a flexible organization [40].

The main identified cell signaling pathways in the adult SSCs were the BMP and the Fibroblast growth factor (FGF) pathways [39,42,44]. Other identified pathways were LIF, Platelet-derived growth factor, glial cell line-derived neurotrophic factor (GDNF), INTEGRIN/TSPAN, and NOTCH1/HES1, which were coming more to expression in undifferentiated SSCs [39].

Furthermore, the undifferentiated SSCs maturated further towards the differentiated spermatogonia state [38]. Inside these cells, the expression of meiotic genes could already be identified, whereas the protein expression was induced in the later primary spermatocyte state [41]. Furthermore, GO terms related to “spermatogenesis”, “meiosis”, “cell cycle”, and “mitosis” were generally identified in differentiating spermatogonia analyzed with scRNA-seq [34,38,42].

Spermatocytes have to undergo meiosis to maturate into spermatids [4]. The different phases of the first meiosis could be distinguished based on the scRNA-seq data [41,42]. The meiotic prophase 1 consists of different succeeding cell states. The leptotene state (which contains three consecutive states) was generally enriched in GO terms such as “DNA metabolic processes” and “chromosome organization”, as well as “meiosis I” [38,42]. Zygotene, pachytene-, and the diplotene state were characterized by the three most significant GO terms from M. Wang et al.: “RNA splicing”, “cell cycle”, and “nuclear division”, respectively. M. Wang et al. gathered the other cell states in the spermatocytes 7 cluster, which was a mixture of diakinesis, metaphase, anaphase, telophase, and the secondary spermatocytes, and the related GO terms reported were “sexual reproduction”, “male gamete generation”, “fertilization”, “cytoskeleton organization”, and “cell wall macromolecule metabolic process” [42]. Notably, the meiotic sex chromosome inactivation could be observed in scRNA-seq data during meiotic prophase 1, with the minimal transcriptional state observed in the pachytene stage [42,50]. Furthermore, the majority of the repressed genes in the X- or Y-chromosome stayed repressed in contrast to a reactivation in the spermatid stage [42].

Spermatids are haploid cells [3] that further undergo a series of morphological changes including a decrease in the spermatid head diameter from 8–10 µm to 5–7 µm, as shown via immunofluorescent staining of testicular tissue [42]. Different papers reported various spermatid states. While J. Guo et al. distinguished two different states of spermatids, the round and the elongated spermatids based on their differential gene expression [38], others identified four or five different spermatids states [41,42], and B. Xia et al. found four substates of round spermatids and four substates of elongated spermatids. ScRNA-seq data showed that the round spermatids had the highest number of genes coming to expression over the other germ- and somatic cells [45], and from the spermatid state onwards, the number of transcripts decreased [42,45]. GO terms such as “gamete generation” and “sexual reproduction” were commonly identified in spermatids. “Cilium movement” and other GO terms related to cell mobility and cytoskeleton were also regularly identified [5,42,50].

Spermatozoa are the cells that will fertilize an oocyte [3]. In the study of J. Guo et al., two spermatozoa states were identified. The upregulated genes associated with the GO terms in the transition from the elongated spermatids towards the first cell cluster of spermatozoa were “ribosome”, “spermatogenesis”, “ubiquitin”, and “sperm mobility/motion”. The downregulated genes were related to “proteolysis”, “sexual reproduction”, “cytoskeleton/centrosome”, “microtubule”, and “mitochondrion”. The maturation from the first spermatozoa cluster to the second was characterized by GO terms of “spermatogenesis”, “ribosome”, “ubiquitin”, “glycolysis”, and “organelle envelope”, and downregulated genes are associated with the GO terms such as “translation elongation”, “structural molecule activity”, “mitochondrion”, “spermatogenesis”, and “microtubule cytoskeleton”. Although two spermatozoa cell clusters are seen, it is suggested that these two clusters are a single population because of a probably different rate in RNA removal/degradation in these already low-RNA-expressing cells. J. Guo et al. suggested that the almost completely repressed gene expression in these cells could be one of the reasons why spermatozoa were difficult to detect via scRNA-seq [38]. J. Guo et al. was the only paper in our selected research articles that separately clustered spermatozoa. However, the repression of gene expression was also observed in the different spermatid states in the studies of M. Wang et al. and B. Xia et al. [42,45].

#### 3.4.2. The Somatic Cell Lineage

The somatic compartment of the adult testis was not frequently undergoing cell division because cell cycle genes in aLCs, SCs, and PTMCs were not dominantly coming to expression [34]. Two other regularly identified cell clusters in the somatic compartment of the testis were macrophages and ECs [34,38,43,44,46,47,49]. Furthermore, it is known that the aLC undergoes three maturating states: the progenitor LC, the immature LC, and the mature LC [60], and Mahyari et al. found in their transcriptomic data that the mature LC was the smallest fraction compared to the immature LC population, which was the largest. The mature LCs were mostly mitotically inactive, whereas about 15% of the immature LCs and around 10% of the progenitor LCs were undergoing cell cycling [48]. Furthermore, the presence of three SC states (Stage_a, Stage_b, and Stage_c) in the adult testis (17–31 years) were identified in the study of Zhao et al. [37]. However, in the results of J. Guo et al., only the mature SC state was detected in the 25-year-old sample [36]. Furthermore, the pleiotropin signaling pathway is well established in the SSCs, the differentiating spermatogonia, and the somatic lineages such as the SCs, LCs, PTMCs, macrophages, ECs, and smooth muscle cells, whereas the ligands (PTN, SDC2, SDC4, and NCL) were coming to expression in the SCs, LCs, and PTMCs [5].

### 3.5. The Aged Testis

#### 3.5.1. The Germ Cell Lineage

The aging of the testis is not similar in each person. Nie et al. could identify two populations in the aged testis: one group with complete spermatogenesis and a second group with an impaired spermatogenesis process from the round spermatid state onwards. In both groups, the State 0 up to State 4 SSCs were identified [49]. The transcriptomics results showed that the BER mechanism and related BER-promoting genes were downregulated, indicating that aged SSCs were more sensitive to de novo mutations. Furthermore, the variation in expression was increased inside the SSCs, differentiating spermatogonia and spermatocytes compared to the spermatids [5].

#### 3.5.2. The Somatic Cell Lineage

The aged testis is morphologically characterized by a decrease in the number of LCs and SCs, the thickening of the basal membrane, increased extracellular matrix (ECM) in the interstitium, and a reduced seminiferous tubular area [5,49]. The transcriptomic data showed that the aged somatic cell lineage is in an inflammatory state, whereas the peptide secretion, hormone response, and growth-related genes were downregulated. In addition to this, K. Xia et al. also determined that LCs and PTMCs had the most age-related transcriptional variation compared to SCs, macrophages, ECs, and smooth muscle cells [5]. Furthermore, Nie et al. found that prominent aLC markers were decreased in both groups of the older testis population (with and without complete spermatogenesis). The LCs of the testis population with the impaired spermatogenesis process brought significantly more LC-PTMC precursor markers to expression compared to the aged testicular tissue with a complete spermatogenesis process [49].

### 3.6. Testicular Cell Culture

The few studies that reported analyses on cultured cells are listed in Table 2.

**Table 2:** Identified papers which performed single-cell RNA-sequencing on cultured cells.

Author	Year of Publication	Age of Cultured Cells	Culture Time	Addition of	Reference
Tan et al.	2020	32 and 37 years old	14 days	MK-2206 HCL	[61]
Liebich et al.	2022	41–48 years old	24 h	FCS	[62]
R. Wang et al.	2022	7 and 15 WPC	24 h	LDN-19318	[29]

MK-2206 HCL = AKT-pathway inhibitor, FCS = fetal calf serum, LDN-19318 = bone morphogenetic protein pathway inhibitor, WPC = weeks post-conception.

The main objectives were to identify molecules influencing the SSC fate towards renewal or differentiation.

R. Wang et al. cultured 7 WPC mitotic GCs and 15 WPC mitotic and mitotic arrested GCs for 24 h using LDN-19318 to inhibit the BMP pathway. ScRNA-seq showed that the expression of ALDH1A2, a retinoic acid (RA)-synthesizing gene, was decreased in 15-WPC-old mitotic arrested GCs after BMP inhibition, indicating that BMP plays a role in the RA expression in later-stage GCs. In addition to this, the BMP pathway inhibition had no effect on the cells because the ALDH1A2 was not expressed in 7-WPC-old cells [29].

Tan et al. cultured spermatogonia from a 32 and a 37-year-old fertile patient for two weeks in different culture conditions. The aim of their study was to find conditions favoring the maintenance of the undifferentiated SSC state, related to State 0 or SSC-1B, identified by their PLPPR3 expression. Cells cultured in media supplemented with FGF2 or FGF2 with an AKT-pathway inhibitor (MK-2206 HCL) were sequenced after two weeks. ScRNA-seq showed that the addition of MK-2206 HCL favored the culture of undifferentiated spermatogonia. Five consecutive clusters of undifferentiated spermatogonia (C1–C5) could be identified based on the presence of undifferentiated spermatogonia marker genes, such as PLPPR3, TSPAN33, and PIWIL4, in combination with a low proportion of differentiating spermatogonia marker genes, such as KIT or STRA8. When cells were cultured in supplemented media with the AKT-pathway inhibitor, 29.7% more cells were seen in C1 compared to C2. In contrast, in the FGF media, the percentages of cells in C1 (20.8%) and C2 (20.9%) were similar. Furthermore, more cells were found in the most undifferentiated cell cluster (C1) after culturing in media containing FGF2 and MK-2206 HCL (36.6%) compared to FGF2 supplementation alone (20.8%). The C1 cluster had GO terms related to undifferentiated SSC, such as “establishment of protein localization to endoplasmic reticulum”, “protein targeting to membrane”, “transcription”, “RNA catabolic process”, and “peptide biosynthetic process”. The C2 cluster downregulated self-renewal and maintenance genes and upregulated genes related to cell differentiation. This study also highlighted other important pathways in the five different SSC clusters. The three most enriched pathways in cell cluster C1 were the EIF2 signaling, mTOR signaling, and regulation of eIF4 and p70S6K signaling pathways. Furthermore, the EIF2 and mTOR signaling pathways were also identified as two of the three main enriched pathways in the C2 cluster together with the phagosome maturation, indicating that these pathways can be important in the maturation of undifferentiated SSCs towards the more differentiated cell clusters (C3–C5). From cluster C3 onwards, the OXPHOS pathway was identified up to cluster C5, where it was also the most enriched pathway of the cell cluster. Unfortunately, this paper did not mention if the enriched pathway determination was based on only the cultured cells in FGF2 media or the cells cultured in the FGF2 media supplemented with the AKT inhibitor [61].

In the study of Liebich et al., PTMCs of two patients aged 41 and 48 years were cultured for 24 h with or without the addition of 10% fetal calf serum (FCS). They compared their data of the PTMCs cultured without FCS to the data from the study of Nie et al. and Di Persio et al. and found an overlap of 58% of the genes between the three datasets. Furthermore, four different cell clusters of PTMCs were found, expressing smooth muscle-related genes and ECM-related genes. Supplementation of the culture medium with FCS induced the proliferation of PTMCs and decreased transcription of the ECM components Decorin and Biglycan. Moreover, PTMCs expressed SSC influencing factors, such as GDNF, C-X-C motif chemokine ligand 12, and nerve growth factor. The expression of these factors was slightly favored in the FCS-supplemented media. The expression of RA-synthesizing genes, ALDH1A1 and ALDH1A3, were upregulated in the PTMCs cultured in the media without FCS, indicating that RA can be formed by this cell type. Additionally, the expression of another RA-synthesizing gene, ALDH1A2, was not detected in PTMCs [62].

### 3.7. Discussion

Different SSC substates have been described among studies. It can be assumed that the earlier defined State 0 by J. Guo et al. [38] is not the most undifferentiated SSC state because two other research groups could define three substates inside the State 0 SSCs [34,47].

ScRNA-seq data induced a paradigm shift in the classification of undifferentiated SSCs [47]. In the past, the undifferentiated SSC pool was classified based on morphological characteristics in the A_dark_ and A_pale_ subpopulations, which were, respectively, described as the reserve stem cells and mitotically active stem cells of the testis [63,64]. Neuhaus et al. was the first to sequence human adult SSCs. They could already confirm, despite their low coverage of the whole transcriptome, that the human SSCs are a heterogeneous cell population [40]. Thus far, the A_dark_ and A_pale_ SSC populations could still not be distinguished from each other based on their gene transcription pattern [36,44,65]. Shami et al. detected in the SPG1 SSC population, which is closely related to the State 0 SSC of J. Guo et al., an overlap of different cell markers between the A_dark_ and A_pale_ populations via immunofluorescence staining [44], and Di Persio et al. found that both the State 0 and the State 1 undifferentiated SSCs can display the A_dark_ morphology [47]. Furthermore, State 0 and 1 SSCs are not in a different phase of the cell cycle [65], supporting the idea that A_dark_ and A_pale_ are the same cell population at different states of the cell cycle [66]. Another previously proposed possibility is that SSCs transition spontaneously between the A_dark_ and A_pale_ [67]. Because several questions remain unanswered, such as how the A_dark_ and A_pale_ populations now relate to the different identified SSC states and if the A_dark_ and A_pale_ populations are in a different cell cycle phase, more research is needed to find a comprehensive answer to this matter. Furthermore, the differentiating SSCs already expressed meiotic genes, which was confirmed via bulk RNA-sequencing performed by Tan et al. These results indicate that this cell population was preparing for downstream events in the following stages of the spermatogenesis process while they also confirm that differentiating spermatogonia still undergo proliferation [61].

With regards to scRNA-seq of SCs, it has been suggested by J. Guo et al. that Stage_a and Stage_b are different physiological states of an immature SC population, but do not represent different developmental states [36]. This theory differs from Zhao et al., who suggested that the maturation of the SC follows three distinct developmental states: Stage_a, Stage_b, and Stage_c [37]. Voigt et al. reanalyzed their own early published data [35] and combined these with data from the studies of Zhao et al. [37] and of J. Guo et al. [36,38]. They reported SCs of Stage_a and b in the prepubertal testis and Stage_c SCs in the peripubertal testis (11, 13, and 14 years old). Furthermore, they could also identify that Stage_c cells also underwent maturation during peripuberty over three other states to reach the fourth or adult SC state [37]. Observations on scRNA-seq of SCs are questionable. Indeed, it is so far unclear why Sohni et al. did not identify SCs in the testicular cell population [34] and why the amount of SCs was also sparse in the 25-year-old patient sample in the study of J. Guo et al. [38], while SCs represent 17.4% of the testicular volume, as earlier defined via histology [68].

It is of note that scRNA-seq regularly showed major modifications around the age of 11 years. This was the age where the LC-PTMC progenitor started to split and where the maturation of SCs towards Stage_c SCs also took place [36,69]. This is in line with the progression from a noncompartmentalized testis containing immature SCs and LC-PTMC precursors towards a well-organized, structured testis with mature SCs, separate aLCs, and PTMCs. SCs become polarized cells due to the formation of the blood–testis barrier (BTB) which formed the SSC niche to control the metabolic environment [70], and the SSCs also start to flatten their shape [35]. Furthermore, OXPHOS was a recurring metabolic pathway identified at this age in SSCs [35] and SCs [69].

Different pathways were identified with scRNA-seq in native testicular tissue over the different developmental states, such as the NOTCH signaling pathway, found in every developmental state [30,34,36,39], the BMP pathway present in the prenatal [29,30], prepuberal [36], and adult testis [39], the KIT pathway detected in the prenatal [30], neonatal [34], and adult testis [49], and the WNT pathway observed from the neonatal phase onwards [34,36,37,39,44]. GDNF, FGF, and Activin were also regularly identified pathways in the prepubertal and adult testis [36,39,42,44]. Moreover, the Activin, KIT, and pleiotropin pathway were downregulated in older male testicular tissue [5,49]. Understanding the activation of such pathways over the developmental stages of the testis is of high value to guide culture media components and to optimize IVM outcomes.

Although many attempts were made in the past, complete IVM of human ITT is not yet optimized to obtain fertilizing-competent spermatozoa. Via scRNA-seq on cultured testicular cells in media, in the presence or absence of an AKT-signaling pathway inhibitor or FCS, the culture requirements for spermatogonia or PTMCs became clearer. Indeed, the transcriptomics results of Tan et al. showed that the inhibition of the AKT-signaling pathway during testicular cell culture favored the maintenance of C1 undifferentiated spermatogonia over the other spermatogonia cell clusters [61]. Based on their results, it can be proposed that stimulating the AKT-signaling pathway via the addition of an AKT-agonist or substrate could favor the differentiation of SSCs to the more developed states.

Tan et al. also highlighted other important pathways in the five different SSC clusters. The EIF2 and mTOR signaling pathways were two of the most enriched pathways found in the C1 and C2 clusters, indicating that these pathways can be important in the maturation of undifferentiated SSCs towards the more differentiated cell clusters (C3–C5). From cluster C3 onwards, the OXPHOS pathway was identified up to cluster C5, where it was also the most enriched pathway of the cell cluster [61]. Based on the identification of metabolic pathways modification in the different cell states identified with scRNA-seq, well-designed culture experiments could overcome knowledge gaps and achieve better IVM outcomes.

(Re)analyzing scRNA-seq can also be an approach to develop hypotheses to optimize IVM of testicular tissue. This was recently performed by Kurek et al. [71]. They reanalyzed data from Li et al. [30] and J. Guo et al. [36] and found in the scRNA-seq data of the pre- and post-natal testis that laminin alpha (LAMA) 1 and collagen type IV were the main components that constructed the basal membrane (BM). Afterwards, they hypothesized that the cell loss in the germ cell lineage during IVM could be due to the disrupted BM composition. After 14 days in vitro culture of post-natal testicular tissue, LAMA1 expression in the seminiferous BM was decreased over time and correlated with the loss of DDX4-positive GCs [71].

This scoping review describes the different developmental stages in a comprehensive way, with a special focus on the GO terms and with the ultimate aim to advance the optimization of IVM of human ITT. While previous papers did not focus on the GO terms, we regularly reported them to describe the cell states. The advantage of using GO terms is having a summarizing and descriptive way to represent different cell states. Using GO terms avoid a summation of gene names without mentioning any functionality or localization, which makes it more accessible to understand the cell condition. Furthermore, genes behind a GO term can be accessed in an online database [72].

Detailed understanding of the spermatogenesis process at a single cell level helps to translate the scRNA-seq data towards clinical applications. As an example, commonly identified cell signaling pathways during the testis development and maturation may highlight factors that can be essential for specific maturation stages during IVM of ITT, which, if successful, will ultimately give the possibility to infertile men to father their own genetic child. Furthermore, an optimized human in vitro model of the testis could be used in the future for drug development and drug screening before clinical administration.

Since the scoping review focused on original scRNA-seq data, papers that only reanalyzed previously published scRNA-seq data were not included. A limitation of the data presented is linked to the early age of scRNA-seq technology and to some technical limitations, as seen for the somatic cell lineage, especially for SC maturation where data remain scarce. Additional studies are therefore needed to distinguish if SCs pass both the SC stage_a and stage_b or not. One of the difficulties to overcome in future scRNA-seq studies is the low proportion of SCs in the analyzed somatic cell population. This low proportion is likely due to the technical limitation of the microfluidic platform that has difficulties with processing large cells, such as SCs, which have a size of ≥40 µm [34,36,43]. A single-nuclei RNA-sequencing could be an alternative to define the transcriptome of the SCs, as nuclei are smaller and will easily fit through the pores of the microfluidic platform.

Another limitation of the data is the complexity of establishing the cadastre of the germ cell lineage during the testis development and maturation. GCs are named differently depending on their differentiation status, proliferation status, and age of the fetus, and there is also heterogeneity between researchers in names of SSC states. The same difficulty was encountered for LCs. While Garcia-Alonso et al. and J. Guo et al. were able to identify and cluster fLCs separately from the other cell types [28,31], other studies annotated the LCs as “Leydig cell precursor” and “differentiated Leydig cell” [30] or “interstitial progenitor cell” [29]. It is, thus, not clear if the latter clusters contain fLCs, aLCs, or a combination of both.

Based on this review of scRNA-seq data, it could be relevant to reconsider in the future the nomenclature of the GCs and the SSCs and to better report which cell type the LC cluster contains.

Additionally, future research should analyze how A_dark_ and A_pale_ cells, observed on histological sections, can be aligned with the discovery of different SSC states.

Caution is required when interpreting scRNA-seq data. Indeed, as normal human testicular tissue samples without any testis-related diseases are sparse, testicular tissue from obstructive azoospermia (OA) patients is commonly used in scRNA-seq experiments [5,34,37,40,41,42,43,45,46,47,48,50]. ScRNA-seq is, thus, frequently performed on samples taken during vasectomy reversals or testicular sperm extraction for downstream fertility purposes [34,41,42,47] and used as control tissue [34,40,43,45,46,47,50], or used undistinguished and combined with data from testicular tissue with a complete spermatogenesis process without any testis-related diseases [5,37,41,42,48]. Although OA patients have a complete spermatogenesis process in their testes [73], including the same cell subtype populations as human healthy testicular tissue [43,46], the transcriptomic profiles can differ [43]. This is supported by the observations of Chen et al. who compared the transcriptomic profiles of OA patients with data from a healthy adult and found that the spermatogenesis process was impaired in OA patients [43]. This study confirmed earlier findings of an impaired spermatogenesis process after vasectomy [74,75]. Furthermore, due to the diversity of the disorders that cause OA, it is likely that the expression profiles coming from OA testes also differ among different individuals.

Excluding papers that used OA samples would result in a low number of remaining papers (*n* = 4), which would not represent the available knowledge of the adult testis. More work is definitely awaited to determine if OA samples are a suitable surrogate or not for sparce adult testis samples without any underlying testicular diseases.

Despite its limitations, scRNA-seq data certainly offers valuable insight into the development and maturation of the testis.

## 4. Conclusions

The scRNA-seq data showed that the testicular cells undergo an extensive maturation process over different ages, although definitions of the various cell states still need to be clarified. In this regard, it is unclear if the histological and morphologically identified A_dark_ and A_pale_ SSCs are actually different cell states and if this classification is surpassed. Furthermore, scRNA-seq presents some technical limitations, especially for SCs, due to the large size of the cells. Single-nuclei RNA-sequencing could be more appropriate in the future to overcome the cell size limitation. Research should be extended to testis samples from healthy fertile men as, although OA patients have a complete spermatogenesis process, the obstruction influences the spermatogenic process and homeostatic balance of the testis. IVM of testicular tissue should recapitulate the passage of both the germ and the somatic cell lineages throughout all the different cell states and morphological changes to eventually obtain haploid, fertilizing-competent spermatozoa with a proper transcriptomic profile. ScRNA-seq on cultured testicular cells suggested that the AKT-signaling pathway is a potential driver of the SSC differentiation and that the involvement of the OXPHOS pathway is dependent on the maturational state of the cells. Regularly identified cell signaling pathways during the testis development and maturation could be worth the investigation and help to identify essential factors required for specific maturation stages in IVM.

## Abbreviations

aLC	Adult Leydig cell
BER	Base-excision repair
BM	Basal membrane
BMP	Bone morphogenetic protein
BTB	Blood–testis barrier
M-CSF, CSF1	Colony stimulating factor 1
EC	Endothelial cell
ECM	Extra cellular matrix
FCS	Fetal calf serum
FGF	Fibroblast growth factor
fLC	Fetal Leydig cell
GC	Germ Cell
GDNF	Glial cell line-derived neurotrophic factor
GEO	Gene Expression Omnibus
GO	Gene Ontology
GSE	GEO series accession number
iLC	Immature Leydig cell
ITT	Immature testicular tissue
IVM	In vitro maturation
LAMA	Laminin alpha
LC	Leydig cell
LC-PTMC	Leydig-peritubular myoid cell
mLC	Mature Leydig cell
OA	Obstructive azoospermia
OXPHOS	Oxidative phosphorylation
pLC	Progenitor Leydig cell
PreSPG	Prespermatogonia
PTMC	Peritubular myoid cell
RA	Retinoic acid
SC	Sertoli cell
scRNA-seq	Single-cell RNA-sequencing
SSC	Spermatogonial stem cell
State f0	Fetal State 0
WPC	Weeks post-conception

## Appendix A

PUBMED

(((“Testis” [Mesh]) OR (“spermatogenesis” [Title/Abstract]) OR (“sertoli cell” [Title/Abstract]) OR (“leydig cell” [Title/Abstract]) OR (“germ-cell” [Title/Abstract]) OR (“germ cell” [Title/Abstract]) OR (“spermatogonia” [Title/Abstract]) OR (“spermatocyte” [Title/Abstract]) OR (“spermatozoa” [Title/Abstract]) OR (“spermatogonial stem cell” [Title/Abstract]) OR (“peritubular myoid cell” [Title/Abstract]))) AND (((“Single-Cell Gene Expression Analysis” [Mesh]) OR (“Single Cell Gene Expression Analysis” [Title/Abstract]) OR (“Single Cell Gene Expression Profiling” [Title/Abstract]) OR (“Single-Cell Gene Expression Profiling” [Title/Abstract]) OR (“Single Cell Transcriptome Analysis” [Title/Abstract]) OR (“transcriptomics” [Title/Abstract]) OR (“Single-Cell Transcriptome Analyses” [Title/Abstract]) OR (“Single-Cell RNA-Seq” [Title/Abstract]) OR (“Single Cell RNA Seq” [Title/Abstract]) OR (“Single-Cell RNA Seq” [Title/Abstract]) OR (“ScRNA-seq” [Title/Abstract]) OR (“ScRNA seq” [Title/Abstract]) OR (“Sc-RNAseq” [Title/Abstract]) OR (“Sc RNAseq” [Title/Abstract]) OR (“ScRNAseq” [Title/Abstract]) OR (“Single-Cell RNAseq” [Title/Abstract]) OR (“Single Cell RNAseq” [Title/Abstract]) OR (“Gene Expression Profilings” [Title/Abstract]) OR (“Transcriptome Profiling” [Title/Abstract]) OR (“Transcriptome Analyses” [Title/Abstract]) OR (“Transcriptome Analysis” [Title/Abstract]) OR (“Gene Expression Analyses” [Title/Abstract]) OR (“Gene Expression Analysis” [Title/Abstract]) OR (“Transcriptome Profilings” [Title/Abstract]))) AND (“human” [All Fields] OR “homo sapiens” [All Fields]).

## Appendix B

EMBASE

(‘testis’/exp OR ‘spermatogenesis’:ti,ab OR ‘sertoli cell’:ti,ab OR ‘leydig cell’:ti,ab OR ‘germ-cell’:ti,ab OR ‘germ cell’:ti,ab OR ‘spermatogonia’:ti,ab OR ‘spermatocyte’:ti,ab OR ‘spermatozoa’:ti,ab OR ‘spermatogonial stem cell’:ti,ab OR ‘peritubular myoid cell’:ti,ab) AND (‘single-cell gene expression analysis’/exp OR ‘Single Cell Gene Expression Analysis’:ti,ab OR ‘Single Cell Gene Expression Profiling’:ti,ab OR ‘Single-Cell Gene Expression Profiling’:ti,ab OR ‘Single Cell Transcriptome Analysis’:ti,ab OR ‘transcriptomics’:ti,ab OR ‘Single-Cell Transcriptome Analyses’:ti,ab OR ‘Single-Cell RNA-Seq’:ti,ab OR ‘Single Cell RNA Seq’:ti,ab OR ‘Single-Cell RNA Seq’:ti,ab OR ‘ScRNA-seq’:ti,ab OR ‘ScRNA seq’:ti,ab OR ‘Sc-RNAseq’:ti,ab OR ‘Sc RNAseq’:ti,ab OR ‘ScRNAseq’:ti,ab OR ‘Single-Cell RNAseq’:ti,ab OR ‘Single Cell RNAseq’:ti,ab OR ‘Gene Expression Profilings’:ti,ab OR ‘Transcriptome Profiling’:ti,ab OR ‘Transcriptome Analyses’:ti,ab OR ‘Transcriptome Analysis’:ti,ab OR ‘Gene Expression Analyses’:ti,ab OR ‘Gene Expression Analysis’:ti,ab OR ‘Transcriptome Profilings’:ti,ab) AND (‘human’ OR ‘homo sapiens’).

## Appendix C

GENE EXPRESSION OMNIBUS DATA SET

((“Testis”[Mesh] OR “spermatogenesis”[DESC] OR “sertoli cell”[DESC] OR “leydig cell”[DESC] OR “germ-cell”[DESC] OR “germ cell”[DESC] OR “spermatogonia”[DESC] OR “spermatocyte”[DESC] OR “spermatozoa”[DESC] OR “peritubular myoid cell”[DESC] OR “spermatogonial stem cell”[DESC]) AND (“Single-Cell Gene Expression Analysis”[DESC] OR “Single Cell Gene Expression Analysis”[DESC] OR “Single Cell Gene Expression Profiling”[DESC] OR “Single-Cell Gene Expression Profiling”[DESC] OR “Single Cell Transcriptome Analysis”[DESC] OR “transcriptomics”[DESC] OR “Single-Cell Transcriptome Analyses”[DESC] OR “Single-Cell RNA-Seq”[DESC] OR “Single Cell RNA Seq”[DESC] OR “Single-Cell RNA Seq”[DESC] OR “ScRNA-seq”[DESC] OR “ScRNA seq”[DESC] OR “Sc-RNAseq”[DESC] OR “Sc RNAseq”[DESC] OR “ScRNAseq”[DESC] OR “Single-Cell RNAseq”[DESC] OR “Single Cell RNAseq”[DESC] OR “Gene Expression Profilings”[DESC] OR “Transcriptome Profiling”[DESC] OR “Transcriptome Analyses”[DESC] OR “Transcriptome Analysis”[DESC] OR “Gene Expression Analyses”[DESC] OR “Gene Expression Analysis”[DESC] OR “Transcriptome Profilings”[DESC]) AND (“Homo sapiens”[Organism] OR “Homo sapiens”[ORGN] OR “Human”[Organism] OR “human”[ORGN])).
